# Psychotherapy in Psychosis: Experiences of Fully Recovered Service Users

**DOI:** 10.3389/fpsyg.2018.01675

**Published:** 2018-09-04

**Authors:** Jone Bjornestad, Marius Veseth, Larry Davidson, Inge Joa, Jan Olav Johannessen, Tor Ketil Larsen, Ingrid Melle, Wenche ten Velden Hegelstad

**Affiliations:** ^1^TIPS – Network for Clinical Research in Psychosis, Stavanger University Hospital, Stavanger, Norway; ^2^Department of Social Studies, Faculty of Social Sciences, University of Stavanger, Stavanger, Norway; ^3^Department of Clinical Psychology, University of Bergen, Bergen, Norway; ^4^School of Medicine and Institution for Social and Policy Studies, Yale University, New Haven, CT, United States; ^5^Network for Medical Sciences, Faculty of Health, University of Stavanger, Stavanger, Norway; ^6^Department of Clinical Medicine, Section of Psychiatry, University of Bergen, Bergen, Norway; ^7^Norwegian Centre for Mental Disorders Research, University of Oslo, Oslo, Norway

**Keywords:** first-episode psychosis, psychosis, schizophrenia, recovery, clinical recovery, psychotherapy

## Abstract

**Background:** Despite the evidence of the importance of including service users’ views on psychotherapy after psychosis, there is a paucity of research investigating impact on full recovery.

**Objectives:** To explore what fully recovered service users found to be the working ingredients of psychotherapy in the recovery process after psychosis.

**Materials and Methods:** The study was designed as a phenomenological investigation with thematic analysis as the practical tool for analysis. Twenty fully recovered service users were interviewed.

**Results:** Themes: (1) Help with the basics, (2) Having a companion when moving through chaotic turf, (3) Creating a common language, (4) Putting psychosis in brackets and cultivate all that is healthy, and (5) Building a bridge from the psychotic state to the outside world.

**Conclusion:** Therapeutic approaches sensitive to stage specific functional challenges seemed crucial for counteracting social isolation and achieving full recovery. Findings indicate that psychotherapy focusing on early readjustment to everyday activities, to what are perceived as meaningful and recovery-oriented, seems to be what is preferred and called for by service users.

## Introduction

In psychosis, standardized treatment guidelines recommend psychotherapy (National Institute for Clinical Excellence [NICE], 2014). Meta-analyses show positive effects both on symptoms and recovery (Lysaker et al., 2010; Jones et al., 2012; Okuzawa et al., 2014), especially for therapies >20 sessions (Sarin et al., 2011). The majority of research is on cognitive behavioral therapy (CBT) (Burns et al., 2014; Hutton and Taylor, 2014). However, meta-analyses have found no clear evidence that CBT is superior to other psychotherapeutic approaches (Tolin, 2010; Jones et al., 2012).

Despite evidence of effectiveness, the factors driving psychotherapeutic change in psychosis remain understudied (Stafford et al., 2013). Research is mainly based on quantitative approaches, and investigations of service-users’ perceptions of therapy are few. In particular, this applies to processes leading to clinical recovery (Davidson et al., 2008), which implies stable symptomatic and functional remission combined (Liberman and Kopelowicz, 2002). Although factors affecting prognosis with regard to clinical recovery are well known, the contextual factors facilitating this form of recovery are less explored. Much of the growing research has focused on service user perspectives on “personal recovery” – on learning how to live a good life with an established mental illness – resulting in a lack of research on factors that might facilitate clinical recovery, particularly from service users’ perspectives (Slade et al., 2012). Given the importance for outcome of therapeutic alliance, and the subjective judgment of its quality, this seems surprising (Horvath et al., 2011). Service user perspectives on recovery-facilitating therapeutic interventions are called for. They could help suggest hypotheses about what constitutes the helpful ingredients in therapy. Further, the ecological validity of large-scale quantitative investigations will benefit from this groundwork to have been properly established.

In this exploratory study, we investigated in what ways service users judged psychotherapeutic interventions to have helped them recover after a first episode psychosis (FEP). This was done by interviewing a sample of 20 persons in clinical recovery, operationalized as symptomatic (Bjornestad et al., 2016b) and functional (Hegelstad et al., 2012) remission throughout the past year.

## Materials and Methods

The study was designed as a phenomenological investigation (Gadamer, 1989; Heidegger, 1996) with thematic analysis (Braun and Clarke, 2006) as the practical tool for analysis. This study consisted of both descriptive and interpretative elements. The descriptive element implies first, that significant knowledge was sought from individuals with lived experience of psychosis, and second, that the central aim was to discover the meaning of such experiences within their broader contexts (Fossey et al., 2002). The interpretative element implies generating data from a reflexive dialog between participants and researchers, and the interviewer checking the mutual understanding of what is being said throughout the interview. This study was approved by the Regional Ethics Committee in Norway (2013/1246-REK sør-øst C). Informed consent was obtained.

### Sample and Recruitment

The sample was recruited from the TIPS-1 study (*N* = 281) and the on-going TIPS-2 study (*N* = 400, approximately), two naturalistic follow-along FEP studies in South-Rogaland, Norway including individuals with FEP from 1997 to 2014. Detailed descriptions of the inclusion criteria and methods have been published elsewhere (Joa et al., 2008; Hegelstad et al., 2012).

Individuals who were included in the study met the following criteria: living in the local catchment area; age 15–65 years; meeting the DSM-IV criteria (as measured by the Structured Clinical Interview for the DSM-IV Axis 1 Disorders) (First et al., 1995) for a first episode of schizophrenia, schizophreniform psychosis, schizoaffective psychosis, delusional disorder, brief psychosis, affective disorder with mood incongruent delusions, or psychosis not otherwise specified; being actively psychotic as measured by the Positive and Negative Syndrome Scale (PANSS) (Kay et al., 1987); not previously having received adequate treatment for psychosis; no neurological or endocrine disorders related to the psychosis; understanding and speaking one of the Scandinavian languages; an IQ over 70; and being able and willing to sign an informed consent. At inclusion participants agreed to baseline assessment, and follow-up after 3 months, and 1, 2, 5, and 10 years.

In this sub-study participants were recruited consecutively at 2-, 5-, and 10-year follow-up. Here, the TIPS team conducted a screening process based on the criteria for clinical recovery. Twenty-seven eligible candidates were contacted; of these, four people refused study participation and three were classified as non-recovered (subsequently after the interview), due to having only 50% part-time work- a criterion being full time employment. Sample size was decided on the basis of stability of findings (Hill et al., 1997), reviewed after 15 and 17 participants. We stopped recruiting after 20 participants, because we considered the last three interviews as not contributing any substantially new information.

The study sample comprised 10 females and 10 males, all ethnic Norwegians. At the interview time-point, they were all living independently and were in full-time employment or education and average years of education after mandatory school (equals high school level) was 1.15 years (range 1–4 years). At baseline, participants’ clinical diagnoses were affective disorder with mood incongruent delusions (*n* = 5); psychosis not otherwise specified (*n* = 8); delusional disorder (*n* = 3); schizoaffective disorder (*n* = 2); and brief psychotic disorder (*n* = 2). The average age at inclusion was 25.8 years (range 17–58 years) and median duration of untreated psychosis was 12 weeks (average 26.5 weeks; range 0–156 weeks). At baseline, sub-study participants showed an equal distribution and severity of psychosis symptoms as compared to the rest of the TIPS-2 sample. The duration of untreated psychosis was significantly shorter among participants compared to non-eligible and non-included subjects of the larger TIPS study. They scored significantly higher on frequency of social meetings and quality of social relations (as measured with Lehman’s Quality of Life Interview) (Lehman, 1996).

At baseline, all participants were recommended to use medication, although seven never used antipsychotic drugs. Twelve participants used low-dose [range 0.04–1.0 defined daily dosage (DDD)] (World Health Organization [WHO], 1996), second-generation antipsychotic medication, mostly Olanzapine, during the first year of treatment. One participant used Perphenazine (0.5 DDD). Eight used medication after 1 year of treatment and six at the time of the interview. All participants received psychotherapy (typically 45 min session) within a few weeks subsequent to baseline registration. Different psychotherapeutic traditions were applied; mostly CBT and modern relational psychodynamic frameworks. On average, participants participated in 76.3 (*range*: 2–416) sessions of psychotherapy, and the average duration was 2.74 (*range*: 0.7–16.0) years. Therapist professions were split between psychologists (*n* = 7), psychiatrists (*n* = 5), psychiatric nurses (*n* = 7), and a social worker (*n* = 1). Thirteen were interviewed at 2 years follow up sessions, 6 at 5 years and 1 at 10 years.

### Measures

Symptom remission was defined in accordance with international standardized criteria (Andreasen, 2006). Individuals were categorized as non-remitted if they reported any relapse, defined as deterioration of symptoms scored >3 on the relevant PANSS scales, during the previous 6 months.

Functional remission was measured by three of the Strauss-Carpenter Level of Function Scale (Strauss and Carpenter, 1977) subscales measuring independent living, role functioning (work, school or full-time homemaking), and social interaction. A score of 0 indicated very poor functioning, while 4 indicated adequate functioning for the total period of the previous 12 months. A score of 4 in all three subscales was required to indicate overall adequate functioning.

Clinical recovery was operationalized as a single variable of “yes” for all patients who met criteria for both symptom remission and adequate functioning.

### Interviews

The first author conducted the interviews between June and December 2014. A semi-structured interview guide was developed in line with the recommendation of Miles et al. (2013) (p. 25), based on the literature on factors facilitating recovery, including psychotherapy (Lysaker et al., 2010; Sarin et al., 2011; Farrelly and Lester, 2014; Okuzawa et al., 2014; Wampold and Imel, 2015) and in collaboration between researchers and two clinically recovered service users. The main aim of the interview was to explore subjectively useful therapeutic components of psychotherapy. The following focus areas guided the interview: (1) person-specific factors; (2) environmental factors; and (3) treatment-related factors. Each theme was introduced with an open-ended question, for example, *how would you describe the treatment you have received, from the day you got difficulties and until today?* The questions were followed-up depending on how much the participant elaborated. Participants were encouraged to relate their experiences to different contexts, asking questions such as, *can you please elaborate on how the psychotherapy helped you in the acute phase?* or *can you tell me a bit more about if and how therapy helped you in your social life?* To capture topics not adequately covered by the interview, participants were invited at the end of each session to provide any information which had not yet been elicited. Pilot interviews were conducted with two clinically recovered service users. Seventeen interviews were conducted at Stavanger University Hospital and three in participants’ homes (*Mean duration*: 51 min; *Range*: 37–76 min). Interviews were audiotaped and transcribed verbatim for the purpose of analysis.

### Analysis

With a particular focus on experiences concerning psychotherapy, we employed a semantic team-based (Binder et al., 2012) thematic analysis (Braun and Clarke, 2006) involving six-steps presented in **Table 1**. As step one in the analytic process and to strengthen the credibility of the study, three of the researchers conducted the six-step procedure independently. Further, during three collaborative meetings the same researchers compared their interpretations, agreed on themes with accompanying quotes, and validated the findings by consensus decision (Hill et al., 1997). The collaborative meetings had a particular focus on steps four to six presented in **Table 1**. To overcome possible disagreement in the analytic process, we agreed on the following decision rules in the preparatory phases of the study: (1) Resolve minor disagreement utilizing the principle of parsimoniousness (i.e., when you have two competing theories that make the same prediction, the simpler one is the more likely). (2) To resolve major disagreement we applied (i) an inductive principle using the raw data as a compass, aiming to select the descriptions most closely reflecting the experience of the phenomena at issue. (ii) Further, we applied the principle of the best argument as described above. Inter-rater agreement between researchers was tested (Pope et al., 2000) and assessed as high.

**Table 1:** Steps of text condensation.

1.	Becoming familiar with the data through thorough reading of the transcribed interviews, forming a main impression of the experiences of the participants, and identification of potential important themes. A theme was defined as a verbalization capturing an important element of the data in relation to the research question, representing a patterned response in the data set.
2.	Generating initial codes, which were defined as the most basic segments of the raw data that could be assessed in a meaningful way regarding the phenomenon.
3.	Searching for and developing candidate themes and sub-themes. Remaining codes were set aside at this phase in a separate category for the purpose of being further analyzed and incorporated when appropriate.
4.	Reviewing themes to develop a coherent thematic map and considering the validity of individual themes in relation to the data set.
5.	Defining and naming themes: Further refining and defining themes, identifying the essence of themes, identifying subthemes and summarizing the contents of the main themes into what each researcher considered to best represent participants’ experiences. When our refinements no longer added substantially to the themes, the analytic process was closed.
6.	To determine the relevance of a particular theme we both counted the frequency of the relevant meaning units combined with our interpretation of how central the theme was perceived to the recovery process.


## Results

The textual analysis resulted in five interrelated themes.

### Help With the Basics

Participants were generally satisfied with their psychotherapy experience. In the acute phase, resulting from high symptom load and functional decline, they perceived themselves as particularly vulnerable. They saw it as crucial that their therapist had a warm and respectful style, and that he or she had specific suggestions and advice how to handle specific issues. For instance, suggestions about how to handle the expectations of others. Suggestions were seen as most helpful when presented in the form of direct and everyday language. Suggestions aimed at daily structures, such as sleep habits, were often seen as particularly useful. These first treatment steps often gave rise to a feeling of safety and hope. Success at this stage was seen as crucial for future treatment adherence.

He (the psychologist) was very intent on normalizing and on establishing a normal daily routine. Getting normal routines for sleeping, having meals… There was a lot of that, I’d say, systemising things. He explained what a psychosis was. Described it as “an earthquake in the brain that takes time to repair.” He gave me advice. He talked as much to me as I to him. He always provided something. If I told him; I’m stuck, I can’t figure it out, then he always had an answer that made me see. He dug things into my head, so that when I actually was in that particular situation in real life then that would pop up. He always explained after he had given me advice. An explanation of what this and that could help with. Yes, and in a way that was meaningful to me. I felt it was useful.

Early reduction of frightening positive symptoms, such as negative voices, was seen as crucial to alleviate pain, and obtain a feeling of mastery. Together with their therapist many participants developed specific strategies to handle positive symptoms. These were assessed as particularly helpful when they contained both a detailed description of the individual symptom phenomenology, *i.e., who the voices belonged to*, and context, *i.e., voices occurred when they were alone in bed*. Being reassured that they already had been handling their positive symptoms gave patients a boost in self-confidence, reduced anxiety and helped them (in their experience) prevent new episodes.

The voices always came at night. So then it was important to try and be ahead of them. Just put on music right away when going to bed, not wait until they appeared because then it would be harder to get rid of them. Or I would wear myself out before bedtime. I was working out quite a lot at the time, so I would have an extra run before I went to bed, in a way, to empty myself completely.

### Having a Companion When Moving Through Chaotic Turf

All participants emphasized that liking their therapist, on a personal level, was crucial for a positive treatment outcome. They valued when the therapist offered unconditional acceptance and genuine closeness. This built trust and helped reduce stress, but also made it easier to address what was difficult. To many, the therapist was seen as a companion: a person they could trust, and to whom they could confide their deepest secrets, including psychotic content and traumatic experiences, which they found shameful. Many participants highlighted that an important role of the therapist was to keep their life story and to maintain a meaningful tie between life events. This was seen as particularly helpful in periods of on-going psychosis and acted as a remedy for mental chaos, disorganization and cognitive deficits, including memory problems.

He took me seriously. I felt he had a caring way about him… With looks, body language, with what he said… He showed that he was present. It was not like a distant look or “let us just get this over with, with her, give her a prescription or something.” It seemed to me that he was really into his job, that he enjoyed his job. There was, like, contact with the eyes and… I understand that you are having a hard time, I want to help… That really meant a lot. I know him and he is a professional, and he has been there for me all along… It may be this, being allowed to be exactly who you are and that there is somebody who understands you and knows your story and has backed you…. He compliments when I do things well, for instance if I got a good grade on an exam, then he would compliment. And we could talk about how I used to be before and… Someone who has kept my story, in a way. Someone I dared talk to.

### Creating a Common Language

Many participants experienced a lack of language for expressing frightening thoughts and emotions, particularly in the initial phase of therapy. This was seen to increase stress and to maintain or even make symptoms worse. Participants described therapy as a language- creating process. Within a frame of stress reduction, focus was usually split between expressing on-going internal states, and expressing oneself prior to when emotions became overwhelming.

I started trusting her, and she was good at asking the right questions and pull things out of me, ask me kind of these a bit scary questions. It was very hard talking about it, but she helped me put things into words. She got me to open up quite a bit… well, the sort of things you don’t say to your parents or friends. It really helped to put things into words. To find out what it was really about… That was scary. The fog lifted a little. I hadn’t had that experience before. I found the missing words, for these thoughts and feelings.

Some participants also explained how delusions became less engrossing and became more manageable through repeated, targeted and corrective conversations about delusional content. Psychotherapy was most often described as the only arena for these types of conversations to take place.

I feel that I got to disentangle the reasons for why I had been behaving as I did. I did have this feeling that someone was tailing me. It was only in therapy that I could talk about this. When I got to say what was going on, things opened up, in a way. I feel that this is what helped me the most. I think that one of the things with having stuff like this going on is that you have to talk it through many times, and then it will sort of encapsulate within a kind of understanding. It is also important to be challenged when you talk about stuff that is really weird. It can help putting things into perspective. Things may not be how you think they are.

### Putting Psychosis in Brackets and Cultivate All That Is Healthy

Many participants appreciated it when their therapist actively reinforced progression and adaptive behavior while at the same time giving less attention to problematic behavior. This approach was emphasized as having helped strengthen everything healthy in the participant. However, a lack of attention to the distressing behaviors could also be perceived as harsh. Hence, to be effective it was seen as a prerequisite that a solid alliance be established and that the intervention be transparently performed, including an explicit explanation of the therapeutic behavior.

I hadn’t had a shower in 3 weeks. She never went into that. But then she has been “on me” as soon as I start pulling myself up, being very supportive about the choices I make. Really nurtured what was healthy. If I said that I feel so depressed, she has said “well, are you, really? Isn’t that a completely natural reaction, being sad, with what you are having to deal with?” She treats me according to how sick I am. It is she who is dynamic according to where I’m at. Because that is often a problem, right, people treating you as if you are ill when you are well, or only seeing the healthy when you are ill. She has pushed me, in a good way. She has never used it against me that I have a lot of skills and stuff, but when I am well, she pushes me to use them.

The approach of distinct and explicit pressure – *i.e., pressured the participants to participate in a social setting when they wished to be socially withdrawn –* was perceived as highly effective and concurrently anxiety provoking. Most participants saw these interventions as crucial to gradually enhancing their ability to handle stress and achieving higher levels of functioning. Similar to the intervention of putting less emphasis on psychosis, this intervention was described as a balancing act, depending on a sensitive therapist knowing when to push, when to hold, and when to take a step back.

He was always very clear about wanting me to be in on it myself. You know, that he couldn’t ”cure” me. He pushed to do work on bad days. In hindsight I can see that this was necessary in order to enable me to handle increasing work loads. It seemed he knew exactly how far he could take that before it would have become too much.

### Building a Bridge From the Psychotic State to the Outside World

Being psychotic was, for most participants, incompatible with regarding oneself as a full citizen. This perception engendered a sense of hopelessness. In order to recover, participants saw it as crucial to break the pattern of passivity and what they perceived as being excluded from the community. Recovery, to them, related to creating a meaningful place in society. Participating in social activities alongside work and school was important. Here, the therapist role was described as a regulating supporter and a consultant; one that could be consulted when needed, but should stay in the background during periods of greater drive, self-esteem and independence. Again, they appreciated their therapist applying sensitive pressure keeping them in a zone of progression, and taking an active role in finding the right workload as well as areas to focus on.

I think really what it is about is finding something meaningful to get out of bed for. Talking to people, talk to a psychologist and get some advice to bring along. Pulling out those pieces of advice when you sit there, alone, feeling down. Use them. That’s the thing. I have become more open after therapy. I have talked to other people. Told at work what was going on, to the project manager and manager. Everything should be focused on functioning in society, not on functioning well in the psychiatric ward.

## Discussion

The aim of this study was to investigate how recovered service users experienced psychotherapy during and after a FEP. Echoing previous studies, findings indicate an early solid psychotherapeutic alliance to be pivotal for a positive course of treatment (Safran and Muran, 2006; Horvath et al., 2011; Priebe et al., 2011). Early therapy was focused on safety and establishing basic structures, while the achievement of full functional recovery and regaining the role of an ordinary citizen was prominent in the later course. Findings add to the existing literature by offering a potential road map for how psychotherapy may catalyze the process of recovery-particularly the path from symptomatic remission into the more protracted stages of achieving full functional recovery.

### Helping Service-Users Recover Using Sensitive Pressure

Participants expressed an explicit desire for a targeted therapy shortly after the acute phase abated. Here, touching the field of self-agency research (Frith, 2014; Bjornestad et al., 2016a), alliance research (Safran and Muran, 2006; Farrelly and Lester, 2014), and consistent with Vygotskys concept of “scaffolding” (Vygotsky, 1980; Davidson et al., 2011) therapists responded by conveying the message that participants had to take the role of active agents in their own change toward recovering. This was combined with systematic and transparent therapeutic pressure reflecting principals of exposure therapy (Hutton and Taylor, 2014). Pressure was seen as most efficient when systematically performed and when pushing the upper limit of the service user’s tolerance. Also, a gradual increase of pressure was perceived as essential to increase tolerance of everyday challenges and to continuously reach higher levels of functioning. Thus, a vigilant and sensitive therapist, constantly adjusting to the increased tolerance of the participant seemed crucial for psychotherapy to be assessed as meaningful and effective.

### Clinical Implications

The approach of early sensitive pressure seemed particularly effective in fighting social withdrawal. However, and in line with systematic reviews and meta-analyses (Farrelly and Lester, 2014; Wampold and Imel, 2015), findings indicate that mutual respect, personal closeness, and perceived therapeutic support were a precondition for the therapist to get into a position of exercising such pressure. This approach thus contrasts with a traditional treatment approach primarily focused on risk, ill health, and disease. Instead, findings indicate that therapy focusing on early readjustment to everyday activities, to what is perceived as meaningful and recovery-oriented, especially increased functioning, seems to be what is preferred and called for by service users.

### Limitations

The main limitation concerns representativeness of the sample. Only two participants fulfilled criteria of core schizophrenia spectrum disorder at the time of the interview. All participants came from an area practicing early detection and intervention, which in itself has been shown to improve course and outcome. Hence, this was a relatively homogeneous group of good prognosis patients. Nevertheless, these patients run the risk of long-term functional disability as well, as the general level of functional recovery in a non-early detection area has been shown to be only 15%. There was, however, heterogeneity present in the study sample with regard to illness history.

A second limitation concerns contamination. This study does not offer the opportunity to single out the perceived helpfulness of therapy versus that of medication. Consistent with previous research (Sohler et al., 2016; Bjornestad et al., 2017), acute phase antipsychotic treatment was mostly perceived as helpful in reducing positive symptoms. However, most participants had stopped using antipsychotics during later course. Also, compared to symptomatic remission, functional remission, not symptom alleviation, was seen as the primary focus of therapy for these participants.

## Author Contributions

JJ, TL, IJ, and WH were involved in funding of TIPS-2. JB, MV, and WH (PI TIPS) contributed to concept development, interviews, performed analyses, and wrote the first draft. All authors have made substantial contributions to all phases of the paper and were involved in study design, provided scientific oversight throughout the project, detailed comments to the paper across several drafts, and edited the paper.

## Conflict of Interest Statement

The authors declare that the research was conducted in the absence of any commercial or financial relationships that could be construed as a potential conflict of interest.
